# Role of Neoadjuvant Therapy for Patients with Adenosquamous Carcinoma of the Pancreas: Outcomes from the National Cancer Database

**DOI:** 10.1007/s12029-025-01269-x

**Published:** 2025-07-02

**Authors:** Amanda K. Walsh, Diamantis I. Tsilimigras, Alex B. Blair, Susan Tsai, Timothy M. Pawlik, Ashish Manne, Shafia Rahman, Eric D. Miller, Kenneth L. Pitter, Jordan M. Cloyd

**Affiliations:** 1 Department of Surgery, The Ohio State University Wexner Medical Center, Columbus, OH 43210 USA; 2 Department of Internal Medicine, Division of Medical Oncology, The Ohio State University Wexner Medical Center, Columbus, OH USA; 3 Department of Radiation Oncology, The Ohio State University Wexner Medical Center, Columbus, OH USA

**Keywords:** Pancreatic adenosquamous carcinoma, Pancreatic cancer, Neoadjuvant therapy, Surgery first, National Cancer Database

## Abstract

**Purpose:**

Pancreatic adenosquamous carcinoma (PASC) is a rare and aggressive form of pancreatic cancer whose management often follows its more common pancreatic ductal adenocarcinoma (PDAC) counterpart. While neoadjuvant therapy (NT) is increasingly utilized prior to surgery for PDAC, whether patients with PASC experience similar benefits is unclear.

**Methods:**

Using the National Cancer Database (NCDB), all patients with stage I-III PASC who underwent surgical resection between 2006 and 2020 were included. Patient and tumor characteristics and overall survival (OS) of patients who underwent surgery first (SF) were compared to those who received NT prior to surgery.

**Results:**

Among 1191 patients with PASC who underwent curative intent resection, 208 (17.5%) received NT, whereas 983 (82.5%) underwent SF. Overall, NT was associated with improved OS compared with an SF approach (median 20.7 vs 15.9 months; *p* = 0.03). On multivariable Cox regression analysis, factors independently associated with improved OS included treatment at an academic/research facility, receipt of NT, and receipt of adjuvant therapy. Factors associated with decreased OS included Black race, positive surgical margins, worse comorbidity score, and higher cancer stage. There was no significant difference in OS between patients who received NT chemotherapy and radiation vs NT chemotherapy alone.

**Conclusion:**

Among patients with localized PASC, the receipt of NT prior to surgical resection was associated with improved OS outcomes. Future research is needed to clarify the optimal neoadjuvant treatment regimen, including the role of preoperative radiation, to enhance response to therapy and improve long-term outcomes.

## Introduction

Pancreatic adenosquamous carcinoma (PASC) is a rare and aggressive form of pancreatic cancer with both glandular and squamous components [1]. The incidence of PASC is estimated to be 1–4% of all exocrine pancreatic malignancies [2]. PASC shares many similarities with pancreatic ductal adenocarcinoma (PDAC), and as a result, its clinical management often follows that of its more common counterpart. Despite the similarities to PDAC, PASC has a worse prognosis with a median overall survival (OS) of less than 6 months for patients with advanced disease and only 11–20 months for those with localized disease who undergo surgical resection [3]. Unfortunately, many patients with PASC are diagnosed at advanced stages with either metastatic or unresectable disease [3, 4].

Neoadjuvant therapy (NT) is increasingly utilized for patients with PDAC [5, 6]. Potential advantages of NT include possible tumor downstaging that leads to subsequent resection, early treatment of micrometastatic disease, and an in vivo assessment of therapy efficacy [7–9]. Additionally, delivering NT prior to surgery increases the rate of complete multimodality therapy since not all patients will be able to receive adjuvant therapy after surgery due to delayed surgical healing or deconditioning [10–12]. While still controversial for patients with resectable PDAC, NT is a preferred treatment approach for patients with borderline resectable (BR) and locally advanced (LA) disease [7, 8, 13, 14]. On the other hand, the role of NT for other periampullary malignancies, including ampullary cancer, distal cholangiocarcinoma, and duodenal cancer, has not been firmly established [15–18].

Due to the rarity of PASC and the recent trends of using NT, there is limited literature on the role of NT for patients with PASC, although retrospective case series and cohort studies suggest that completing multimodality therapy is associated with improved outcomes [19, 20]. Whether similar benefits of NT observed in patients with PDAC can be expected in patients with localized PASC remains unknown. Therefore, the objective of the current study was to assess the impact of NT prior to surgery on OS for patients with localized PASC. Given that PASC is thought to be particularly radiation-sensitive, the utility of radiation as a component of NT was also explored.

## Methods

### Study Population

Using the National Cancer Database (NCDB), all patients with stage I-III adenosquamous carcinoma of the pancreas (International Classification of Diseases for Oncology codes C25.0–C25.3, C25.7–C25.9, and histology code 8560) who underwent pancreatectomy (Surgery of the Primary Site Codes 30–80) between 2006 and 2020 were included. The National Cancer Database (NCDB) is a nationwide clinical oncology database that contains data from over 1500 Commission on Cancer accredited facilities [21, 22]. Patients with other histology and individuals who underwent other procedures were excluded.

The following sociodemographic, clinical, and tumor data included in the NCDB were considered for this study: age at diagnosis, sex, race, insurance status, year of diagnosis, household income, treating facility type, distance from the treating facility, time to treatment initiation, tumor grade/differentiation, surgical margins, Charlson–Deyo comorbidity score, NCDB analytic stage group, clinical stage group, clinical T stage, clinical N stage, pathologic stage group, pathologic T stage, pathologic N stage, length of neoadjuvant treatment, receipt of neoadjuvant therapy, type of neoadjuvant chemotherapy, and receipt of adjuvant therapy.

For the purposes of this study, age at diagnosis was reported in years. Sex was reported as male or female. Patient race was classified as White, Black, and other/unknown. Insurance status was reported as private, government (Medicare, Medicaid, and Other Government), and unknown/not insured. Year of diagnosis was reported in terciles (2006–2010, 2011–2015, and 2016–2020). Household income was grouped by 2008–2012 quartiles and reported as less than $38,000, $38,000–47,999, $48,000–62,999, and $63,000 or more. Facility type was categorized as Comprehensive/Community Cancer Program, Academic/Research Program, and Integrated Network Cancer Program based on the primary treating facility when treatment was fragmented [23]. Distance from treating facility was categorized in miles as 0–49, 50–99, > 100, and unknown. Grade/differentiation was categorized as well, moderate, poor, anaplastic, and not determined. Surgical margins were categorized as R0 and R1/R2/unknown. Charlson–Deyo comorbidity score was reported as 0–1 and ≥ 2. Neoadjuvant therapy and adjuvant therapy were classified as neither, chemotherapy only, radiation therapy only, and chemotherapy and radiation therapy. Chemotherapy was classified as single agent, multiagent, and unknown. NT was determined by either reported treatment sequence or treatment initiation timing.

### Statistical Analysis

Patients who underwent surgery first (SF) were directly compared with those who received NT prior to surgical resection. The NT group included patients who underwent neoadjuvant chemotherapy, neoadjuvant radiation, and neoadjuvant chemotherapy and radiation. Continuous variables were reported as medians with interquartile ranges and compared using the Mann–Whitney *U* test. Trends in the use of NT over time were calculated using Kendall’s tau *b*. Categorical variables were reported as totals and percentages and compared using the *χ*^2^ or Fisher’s exact tests, as appropriate. OS was the primary outcome and was defined as the time interval from the date of initial diagnosis until death or last follow-up. OS was censored at the date of last follow-up for living patients. OS was estimated using the Kaplan–Meier method and compared between groups using the log-rank test. Cox proportional hazards regression analysis was used to evaluate associations between patient, tumor, and hospital characteristics and OS. Regression coefficients were reported as hazard ratios (HRs) and corresponding 95% confidence intervals. Variables with *p* values less than 0.10 on univariable analysis were included in multivariable analysis. A *p* value of less than 0.05 was considered statistically significant. A subset of patients treated with neoadjuvant chemotherapy versus neoadjuvant chemoradiation was compared as above. All statistical analysis was performed with IBM SPSS Statistics Version 29.0.2.0.

## Results

**Fig. 1:**
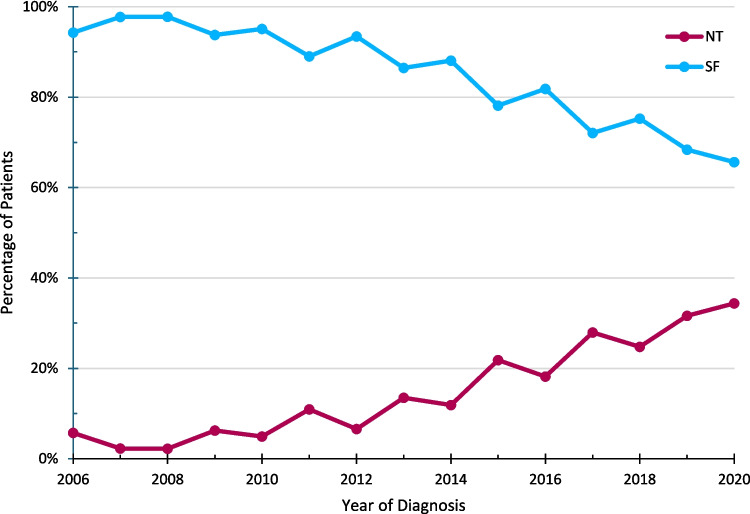
Percentage of patients treated with NT versus SF from 2006 to 2020

Among 1191 patients with PASC who underwent curative intent resection, 208 (17.5%) received NT, whereas 983 (82.5%) underwent SF. For patients who received NT, 156 (75.0%) received preoperative chemotherapy alone, 49 (23.6%) received chemotherapy and radiation therapy, and 3 (1.4%) received radiation therapy alone. The median length of NT was 119 days (IQR 91–163); 15 (7.3%) patients received single-agent therapy, 183 (89.3%) received multiagent therapy, and 7 (3.4%) were treated with an unrecorded agent. There was an increase in the use of NT over the study period with 11 (5.3%) patients in 2006–2010, 60 (28.8%) of patients in 2011–2015, and 137 (65.9%) of patients in 2016–2020 (*τb* = 0.222, *p* < 0.001, Fig. 1). On Kaplan–Meier analysis for the overall cohort, median OS did not significantly change during the study period (2006–2010: 15.9 months, 2011–2015: 16.1 months, and 2016–2020: 17.8 months; *p* = 0.17).

Table 1 reports the clinicopathologic characteristics of patients receiving NT compared with individuals undergoing SF. Patients who received NT were younger (median NT 67 [59,73] vs SF 69 [61,76] years, *p* = 0.007) more likely to be white (*p* = 0.03), to be diagnosed in a more recent year (*p* < 0.001), to have higher household income (*p* = 0.009), to be treated at an academic/research facility (*p* = 0.002), to experience longer time to treatment initiation (median NT 26 [18, 36] vs SF 18 [4, 24] days, *p* < 0.001), and to have stage I disease (*p* < 0.001). NT patients were also more likely to have a worse Charleson-Deyo comorbidity score (*p* < 0.001), live farther from their treatment facility (*p* < 0.001), and were less likely to receive any adjuvant therapy (*p* < 0.001) compared with SF patients.

**Table 1:** Patient and tumor characteristics of the total cohort of patients with PASC who received NT vs SF

Variable	Full cohort (*N* = 1191)		Surgery first (*N* = 983)		Neoadjuvant therapy (*N* = 208)		*p* value
	Count	%	Count	%	Count	%	
Age at diagnosis (median, IQR)	68	(61, 75)	69	(61, 76)	67	(59, 73)	0.007
Sex							
Male	640	53.7%	532	54.1%	108	51.9%	0.564
Female	551	46.3%	451	45.9%	100	48.1%	
Race							
White	1010	84.8%	821	83.5%	189	90.9%	0.027
Black	116	9.7%	104	10.6%	12	5.8%	
Other/unknown	65	5.5%	58	5.9%	7	3.4%	
Insurance status							
Private	406	34.1%	335	34.1%	71	34.1%	0.065
Government	760	63.8%	623	63.4%	137	65.9%	
Unknown/not insured	25	2.1%	25	2.5%	0	0.0%	
Year of diagnosis							
2006–2010	249	20.9%	238	24.2%	11	5.3%	< 0.001
2011–2015	441	37.0%	381	38.8%	60	28.8%	
2016–2020	501	42.1%	364	37.0%	137	65.9%	
Household Income							
Less than $38,000	145	14.1%	130	15.1%	15	9.0%	0.009
$38,000–$47,999	181	17.6%	162	18.8%	19	11.4%	
$48,000–$62,999	267	25.9%	218	25.3%	49	29.5%	
$63,000 or more	436	42.4%	353	40.9%	83	50.0%	
Facility type							
Comprehensive/Community Cancer Program	304	25.7%	271	27.7%	33	15.9%	0.002
Academic/Research	686	57.9%	547	56.0%	139	67.1%	
Integrated Cancer Network	194	16.4%	159	16.3%	35	16.9%	
Distance from treating facility							
0–49 miles	813	68.5%	694	71.0%	119	57.2%	< 0.001
50–99 miles	108	9.1%	89	9.1%	19	9.1%	
100 + miles	104	8.8%	76	7.8%	28	13.5%	
Unknown	161	13.6%	119	12.2%	42	20.2%	
Days to treatment (median, IQR)	19	(6, 32)	18	(4, 30)	26	(18, 36)	< 0.001
Grade/differentiation							
Well differentiated	8	0.9%	8	1.0%	0	0.0%	< 0.001^a^
Moderately differentiated	252	28.0%	226	29.0%	26	21.7%	
Poorly differentiated	498	55.3%	450	57.7%	48	40.0%	
Undifferentiated; anaplastic	13	1.4%	12	1.5%	1	0.8%	
Unknown	129	14.3%	84	10.8%	45	37.5%	
Surgical margins							
R0	915	76.8%	752	76.5%	163	78.4%	.589
R1/R2/unknown	276	23.2%	231	23.5%	45	21.6%	
Charlson–Deyo score							
0–1	1058	88.8%	876	89.1%	182	87.5%	< 0.001^a^
≥ 2	133	11.2%	107	10.9%	26	12.5%	
NCDB analytic stage group							
Stage I	184	15.4%	126	12.8%	58	27.9%	< 0.001
Stage II	908	76.2%	770	78.3%	138	66.3%	
Stage III	99	8.3%	87	8.9%	12	5.8%	
Clinical T stage							
0	4	0.3%	3	0.3%	1	0.5%	< 0.001
1	93	7.8%	83	8.4%	10	4.8%	
2	422	35.4%	349	35.5%	73	35.1%	
3	397	33.3%	301	30.6%	96	46.2%	
4	47	3.9%	23	2.3%	24	11.5%	
Unknown	228	19.1%	224	22.8%	4	1.9%	
Clinical N stage							
0	772	64.8%	633	64.4%	139	66.8%	< 0.001^a^
1	208	17.5%	149	15.2%	59	28.4%	
2	5	0.4%	4	0.4%	1	0.5%	
Unknown	206	17.3%	197	20.0%	9	4.3%	
Pathologic T stage							
0	4	0.3%	1	0.1%	3	1.4%	< 0.001
1	30	2.5%	21	2.1%	9	4.3%	
2	202	17.0%	185	18.8%	17	8.2%	
3	790	66.3%	701	71.3%	89	42.8%	
4	38	3.2%	35	3.6%	3	1.4%	
Unknown	127	10.7%	40	4.1%	87	41.8%	
Pathologic N stage							
0	416	34.9%	368	37.4%	48	23.1%	< 0.001
1	599	50.3%	527	53.6%	72	34.6%	
2	47	3.9%	46	4.7%	1	0.5%	
Unknown	129	10.8%	42	4.3%	87	41.8%	
Adjuvant therapy							
None	455	38.2%	329	33.5%	126	60.6%	< 0.001
Chemotherapy	530	44.5%	469	47.7%	61	29.3%	
Radiation therapy	15	1.3%	10	1.0%	5	2.4%	
Chemotherapy and radiation therapy	191	16.0%	175	17.8%	16	7.7%	

^a^Fisher’s exact test

**Fig. 2:**
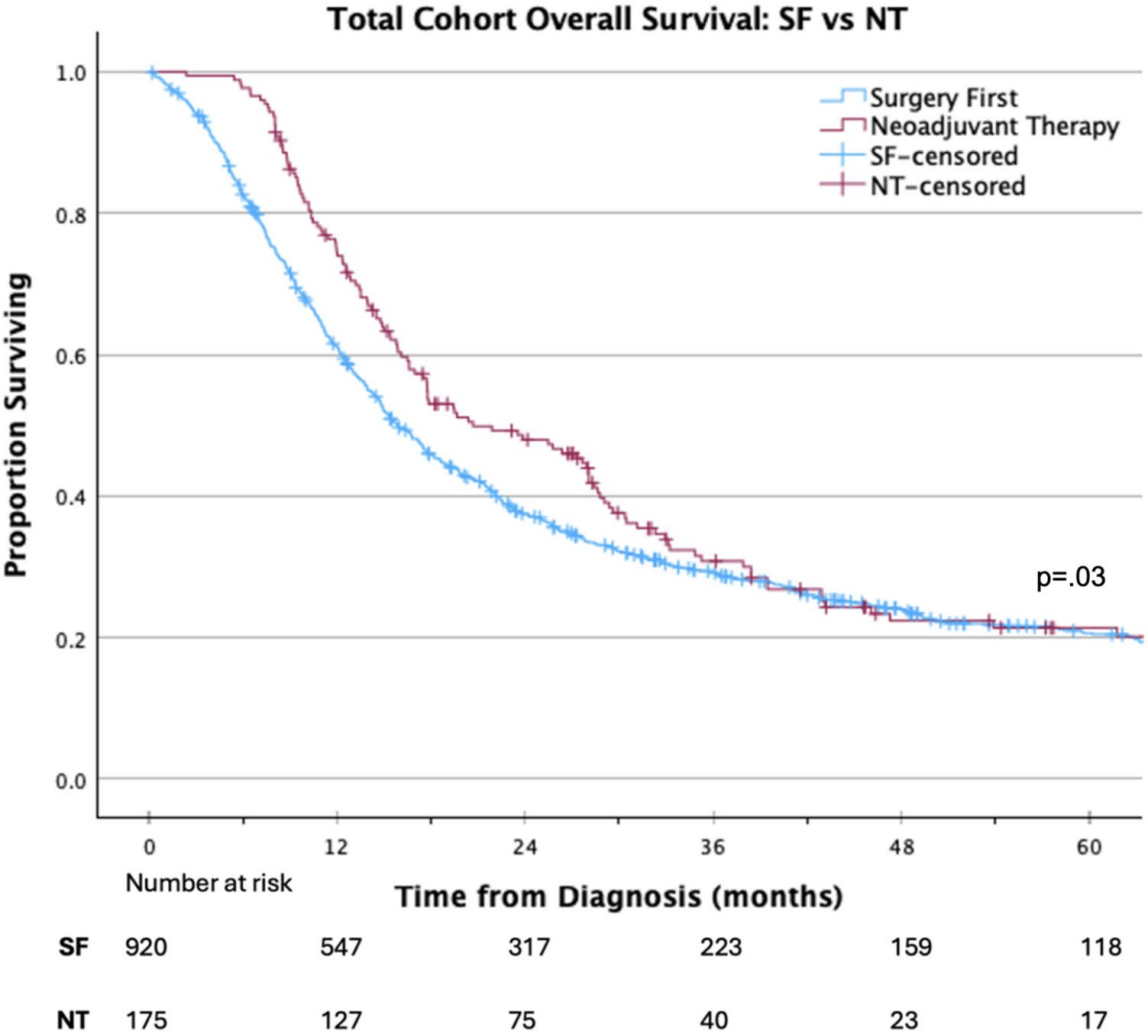
Overall survival of patients with pancreatic adenosquamous carcinoma receiving NT versus SF

On Kaplan Meier analysis, NT was associated with improved OS compared with a SF approach (median 20.7 vs 15.9 months; *p* = 0.03; Fig. 2). On multivariable Cox regression analysis, factors independently associated with improved OS included: treatment at an academic/research facility (HR 0.84 95% CI 0.71–0.98, *p* < 0.05), receipt of NT (HR 0.74 95% CI 0.61–0.91, *p* < 0.05), and receipt of adjuvant therapy (HR 0.56 95% CI 0.51–0.68, *p* < 0.001). Factors associated with decreased OS included black race (HR 1.32 95% CI 1.06–1.64, *p* < 0.05), positive surgical margins (HR 1.54 95% CI 1.31–1.80, *p* < 0.001), worse Charlson–Deyo comorbidity score (HR 1.33 95% CI 1.07–1.67, *p* < 0.05), and NCDB analytic stage groups II and III (HR 1.65 95% CI 1.32–2.07, *p* < 0.001 and HR 2.56 95% CI 1.86–3.52, *p* < 0.001, respectively) (Table 2).

**Table 2:** Cox proportional hazards analysis for overall survival in the total cohort

Variable	Univariate analysis, HR (95% CI)	*p* value	Multivariate analysis, HR (95% CI)	*p* value
Age at diagnosis	1.00 (1.00–1.01)	0.168		
Sex				
Male	Ref		Ref	
Female	0.89 (0.78–1.02)	0.100	0.89 (0.77–1.02)	0.084
Race				
White	Ref		Ref	
Black	1.30 (1.04–1.61)	0.020	1.32 (1.06–1.64)	**0.015**
Other/unknown	0.73 (0.53–1.02)	0.065	0.79 (0.56–1.11)	0.169
Insurance status				
Private	Ref		Ref	
Government	1.07 (0.92–1.23)	0.384	0.98 (0.84–1.13)	0.751
Unknown/not insured	1.51 (0.97–2.35)	0.070	1.39 (0.89–2.19)	0.149
Year of diagnosis				
2006–2010	Ref		Ref	
2011–2015	0.94 (0.79–1.11)	438	1.01 (0.85–1.20)	0.897
2016–2020	0.84 (0.70–1.01)	0.066	0.96 (0.79–1.16)	0.673
Household income				
Less than $38,000	Ref			
$38,000–$47,999	1.07 (0.84–1.38)	0.575		
$48,000–$62,999	1.02 (0.81–1.29)	0.869		
$63,000 or more	0.90 (0.73–1.12)	0.358		
Facility type				
Comprehensive/Community Cancer Program	Ref		Ref	
Academic/Research	0.85 (0.73–1.00)	0.048	0.84 (0.71–0.98)	**0.032**
Integrated Cancer Network	0.92 (0.75–1.14)	0.465	0.88 (0.71–1.09)	0.238
Distance from treating facility				
0–49 miles	Ref			
50–99 miles	0.95 (0.75–1.20)	0.952		
100 + miles	0.91 (0.71–1.16)	0.911		
Unknown	0.83 (0.67–1.02)	0.831		
Grade/differentiation				
Well differentiated	Ref			
Moderately differentiated	0.70 (0.33–1.49)	0.702		
Poorly differentiated	0.94 (0.45–1.99)	0.940		
Undifferentiated; anaplastic	0.67 (0.25–1.75)	0.667		
Unknown	0.81 (0.37–1.73)	0.805		
Surgical margins				
R0	Ref		Ref	
R1/R2/unknown	1.59 (1.36–1.86)	< 0.001	1.54 (1.31–1.80)	** < 0.001**
Charlson–Deyo score				
0–1	Ref		Ref	
≥ 2	1.37 (1.10–1.70)	0.005	1.33 (1.07–1.67)	**0.012**
NCDB analytic stage group				
Stage I	Ref		Ref	
Stage II	1.65 (1.33–2.06)	< 0.001	1.65 (1.32–2.07)	** < 0.001**
Stage III	2.82 (2.06–3.85)	< 0.001	2.56 (1.86–3.52)	** < 0.001**
Neoadjuvant therapy				
No	Ref		Ref	
Yes	0.81 (0.67–0.98)	0.033	0.74 (0.61–0.91)	**0.005**
Adjuvant therapy				
No	Ref		Ref	
Yes	0.66 (0.57–0.75	< 0.001	0.59 (0.51–0.68)	**< 0.001**

*P* values <.05 on multi-variable Cox analysis are bolded

**Fig. 3:**
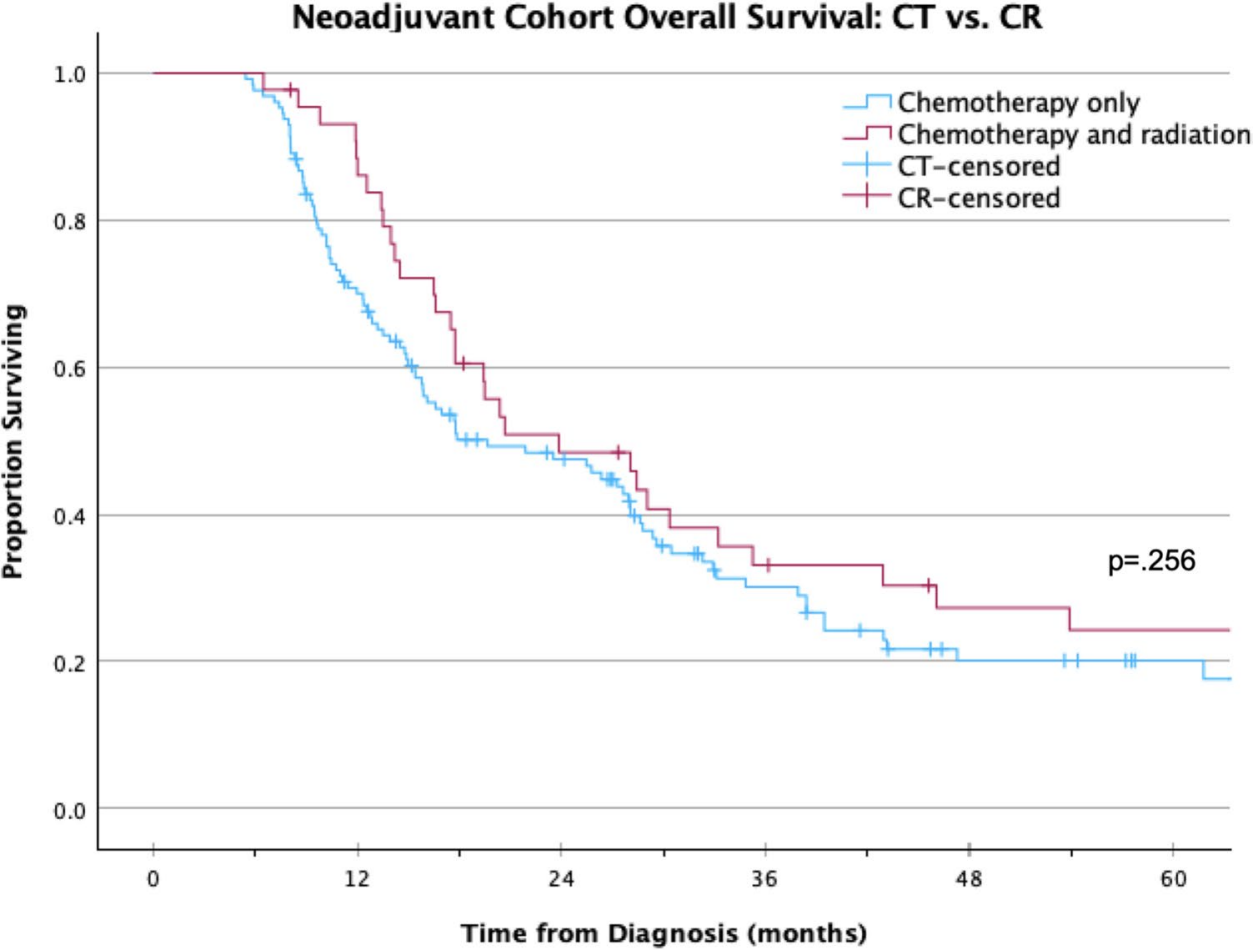
Overall survival outcomes comparing patients treated with NT chemotherapy versus NT chemotherapy and radiation

Table 3 compares patients who received neoadjuvant chemotherapy to those who received neoadjuvant radiation and chemotherapy, both sequential and concurrent. Patients who received neoadjuvant chemotherapy were more likely to be treated in a more recent year and more likely to have a moderately differentiated tumor compared with those who received neoadjuvant chemotherapy and radiation. On Kaplan–Meier analysis, there was no significant difference in OS between patients receiving neoadjuvant chemotherapy versus neoadjuvant chemotherapy and radiation therapy (median 19.7 vs 23.9 months, respectively; *p* = 0.256; Fig. 3). On multivariable Cox regression analysis of the NT cohort, neoadjuvant radiation was not associated with OS whereas receipt of both adjuvant chemotherapy and radiation therapy (HR 0.34 95% CI 0.17–0.67, *p* < 0.01) and R1/2 surgical margins were (HR 1.94 95% CI 1.24–3.05, *p* < 0.01, Table 4).

**Table 3:** Patient and tumor characteristics of the NT subset cohort of patients with PASC who received NT chemotherapy vs NT chemotherapy and radiation

	All NT (*N* = 205)		NT chemotherapy (*N* = 156)		NT chemotherapy and radiation (*N* = 49)		*p* value
Variable	Count	%	Count	%	Count	%	
Age at diagnosis (median, IQR)	67	(59, 73)	68	(60, 74)	62	(56, 71)	0.065
Sex							
Male	108	52.7%	84	53.8%	24	49.0%	0.552
Female	97	47.3%	72	46.2%	25	51.0%	
Race							
White	186	90.7%	139	89.1%	47	95.9%	0.509^a^
Black	12	5.9%	11	7.1%	1	2.0%	
Other/unknown	7	3.4%	6	3.8%	1	2.0%	
Insurance status							
Private	70	34.1%	48	30.8%	22	44.9%	0.069
Government	135	65.9%	108	69.2%	27	55.1%	
Unknown/not insured	0	0.0%	0	0.0%	0	0.0	
Year of diagnosis							
2006–2010	10	4.9%	3	1.9%	7	14.3%	< 0.001
2011–2015	60	29.3%	41	26.3%	19	38.8%	
2016–2020	135	65.9%	112	71.8%	23	46.9%	
Household income							
Less than $38,000	15	9.1%	13	10.5%	2	4.9%	0.531^a^
$38,000–$47,999	19	11.5%	14	11.3%	5	12.2%	
$48,000–$62,999	48	29.1%	33	26.6%	15	36.6^	
$63,000 or more	83	50.3%	64	51.6%	19	46.3%	
Facility type							
Comprehensive/Community Cancer Program	32	15.7%	29	18.7%	3	6.1%	0.107
Academic/Research	138	67.6%	101	65.2%	37	75.5%	
Integrated Cancer Network	34	16.7%	25	16.1%	9	18.4%	
Distance from treating facility							
0–49 miles	118	57.6%	88	56.4%	30	61.2%	0.680
50–99 miles	19	9.3%	16	10.3%	3	6.1%	
100 + miles	28	13.7%	20	12.8%	8	16.3%	
Unknown	40	19.5%	32	20.5%	8	16.3%	
Grade/differentiation							
Well differentiated	0	0.0%	0	0.0%	0	0.0%	0.008^a^
Moderately differentiated	26	22.0%	24	28.2%	2	6.1%	
Poorly differentiated	47	39.8%	29	34.1%	18	54.5%	
Undifferentiated; anaplastic	1	0.8%	0	0.0%	1	3.0%	
Unknown	44	37.3%	32	37.6%	12	36.4%	
Surgical margins							
R0	161	78.5%	118	75.6%	43	87.8%	0.076
R1/R2/unknown	44	21.5%	38	2.4%	6	12.2%	
Charlson–Deyo score							
0–1	179	87.3%	135	86.5%	44	89.8%	0.559
≥ 2	26	12.7%	21	13.5%	5	10.2%	
NCDB analytic stage group							
Stage I	57	27.8	41	26.3%	16	32.7%	0.044
Stage II	136	66.3%	109	69.9%	27	55.1%	
Stage III	12	5.9%	6	3.8%	6	12.2%	
Clinical T Stage							
0	1	0.5%	0	0.0%	1	2.0%	.007^a^
1	10	4.9%	7	4.5%	3	6.1%	
2	71	34.6%	60	38.5%	11	22.4%	
3	95	46.3%	73	46.8%	22	44.9%	
4	24	11.7%	12	7.7%	12	24.5%	
Unknown	4	2.0%	4	2.6%	0	0.0%	
Clinical N stage							
0	138	67.3%	105	67.3%	33	67.3%	0.373^a^
1	57	27.8%	43	27.6%	14	28.6%	
2	1	0.5%	0	0.0%	1	2.0%	
Unknown	9	4.4%	8	5.1%	1	2.0%	
Pathologic T stage							
0	3	1.5%	0	0.0%	3	6.1%	0.023^a^
1	9	4.4%	7	4.5%	2	4.1%	
2	17	8.3%	10	6.4%	7	14.3%	
3	89	43.4%	72	46.2%	17	34.7%	
4	3	1.5%	2	1.3%	1	2.0%	
Unknown	84	41.0%	65	41.7%	19	38.8%	
Pathologic N stage							
0	48	23.4%	27	17.3%	21	42.9%	< 0.001^a^
1	72	35.1%	63	40.4%	9	18.4%	
2	1	0.5%	1	0.6%	0	0.0%	
Unknown	84	41.0%	65	41.7%	19	38.8%	
Adjuvant therapy							
None	125	61.0%	81	86.5%	44	89.8%	0.114^a^
Chemotherapy	59	28.8%	54	34.6%	5	10.2%	
Radiation therapy	5	2.4%	5	3.2%	0	0.0%	
Both chemotherapy and radiation therapy	16	7.8%	16	10.3%	0	0.0%	

^a^Fisher’s exact test

**Table 4:** Cox proportional hazards analysis for overall survival in the NT subgroup cohort

Variable	Univariate analysis, HR (95% CI)	*p* value	Multivariate analysis, HR (95% CI)	*p* value
Age at diagnosis	1.01 (1.00–1.03)	0.107		
Sex				
Male	Ref			
Female	0.86 (0.57–1.31)	0.484		
Race				
White	Ref			
Black	1.27 (0.64–2.50)	0.493		
Other/unknown	0.51 (0.16–1.61)	0.252		
Insurance status				
Private	Ref			
Government	1.10 (0.76–1.61)	0.605		
Year of diagnosis				
2006–2010	Ref			
2011–2015	1.06 (0.50–2.25)	0.874		
2016–2020	1.09 (0.52–2.30)	0.811		
Household income				
Less than $38,000	Ref			
$38,000–$47,999	0.68 (0.27–1.66)	0.393		
$48,000–$62,999	1.07 (0.52–2.20)	0.845		
$63,000 or more	0.95 (0.48–1.88)	0.887		
Facility type				
Comprehensive/Community Cancer Program	Ref		Ref	
Academic/Research	0.86 (0.53–1.39)	0.542	1.03 (0.63–1.68)	0.932
Integrated Cancer Network	0.47 (0.24–0.94)	0.032	0.58 (0.29–1.16)	0.126
Distance from treating facility				
0–49 miles	Ref			
50–99 miles	0.78 (0.42–1.44)	0.424		
100 + miles	0.67 (0.38–1.17)	0.159		
Unknown	0.86 (0.54–1.36)	0.514		
Grade/differentiation				
Moderately differentiated	Ref			
Poorly differentiated	1.55 (0.89–2.70)	0.124		
Undifferentiated; anaplastic		0.970		
Unknown	1.35 (0.76–2.39)	0.307		
Surgical margins				
R0	Ref		Ref	
R1/R2/unknown	1.52 (1.00–2.30)	0.049	1.63 (1.06–2.52)	**0.027**
Charlson–Deyo score				
0–1	Ref			
≥ 2	1.14 (0.63–2.07)	0.662		
NCDB analytic stage group				
Stage I	Ref		Ref	
Stage II	1.70 (1.06–2.73)	0.029	1.45 (0.88–2.37)	0.114
Stage III	1.61 (0.65–4.00)	0.305	1.19 (0.47–3.00)	0.719
Neoadjuvant therapy				
Chemotherapy	Ref		Ref	
Chemotherapy and radiation	0.79 (0.53–1.19)	0.258	0.71 (0.45–1.12)	0.141
Adjuvant therapy				
None	Ref		Ref	
Chemotherapy	0.75 (0.50–1.14)	0.179	0.70 (0.45–1.09)	0.119
Chemotherapy and radiation	0.47 (0.23–0.98)	0.043	0.26 (0.12–0.58)	** < 0.001**

*P* values <.05 on multi-variable Cox analysis are bolded

## Discussion

PASC is an aggressive malignancy with a poor prognosis [1–3]. Multimodal therapy, including chemotherapy, surgical resection, and radiation therapy when applicable, leads to optimal outcomes for patients with localized cancers [5, 16, 25, 26]. While the delivery of chemotherapy and/or radiation therapy prior to surgical resection is increasingly used for patients with PDAC, the role of NT for PASC has been understudied [13, 14, 19, 25]. In this retrospective review of the NCDB, NT prior to surgical resection was associated with improved OS compared with SF even after controlling for confounding factors. Given the limitations associated with a retrospective cancer registry study without intention-to-treat data, as well as the challenges in clinically diagnosing PASC, additional research on optimal treatment sequencing is needed for this aggressive malignancy.

Previous studies have emphasized the importance of multimodality therapy for PASC. Hue et al. utilized the NCDB and reported that receipt of chemotherapy, either in a neoadjuvant or adjuvant manner, was associated with improved OS [19]. Those results build upon other studies that suggest that surgery and chemotherapy are both associated with improved outcomes compared with no treatment and highlight that the best long-term outcomes are observed in patients who are able to receive both treatments as is true for patients with PDAC [3, 4, 27]. Given its relative rarity and in the absence of prospective evidence, current treatment recommendations for PASC are largely based on guidelines for PDAC. Based on several randomized controlled trials, meta-analyses, and expert opinion, NT is now the preferred option for BR and LA PDAC and an acceptable option for potentially resectable PDAC [4, 7, 13, 14, 19, 25, 28].

To the best of our knowledge, the current study is the first to specifically evaluate the role of NT for PASC. Notwithstanding its limitations, given the similarities of our results to prior NCDB studies conducted in patients with PDAC, our findings suggest that the role of NT in PASC might be considered similarly. This is important since NT is often prescribed based on a fine needle aspiration biopsy which may be insufficient to firmly diagnose PASC. In addition to appearing like PDAC on imaging, a threshold of greater than 30% squamous component is used to formally diagnose PASC, but the glandular components are not often equally distributed throughout a tumor [1, 20]. Therefore, many PASC patients may have been treated for presumed PDAC and only diagnosed retrospectively after surgery. When considered with the rarity of PASC and these diagnostic challenges, our finding that the role of NT for PASC may be similar to that of PDAC is reassuring.

A recent study noted that an increased squamous component of PASC is associated with more aggressive behavior and worse outcomes [29]. Radiation therapy is a key treatment component of other squamous histologies, but its importance in treating the squamous component of PASC has not been thoroughly explored and remains controversial [9, 20, 29, 30]. Some studies have suggested that adjuvant chemoradiation may confer a survival benefit for patients with PASC [20]. For that reason, we conducted a subset analysis comparing patients who received neoadjuvant chemotherapy with those who received both neoadjuvant chemotherapy and radiation which revealed no significant difference in OS. Interestingly, among patients receiving NT, the receipt of adjuvant radiation was independently associated with improved OS, suggesting this treatment modality should continue to be investigated in PASC. On the other hand, given the limitations of this retrospective study, this finding could be related to inherent selection biases between patients who were offered adjuvant chemoradiation after NT and surgery and those who were not. Given the mixed literature on the role of adjuvant chemoradiation for both PDAC and PASC, additional research is needed to clarify the role of neoadjuvant and adjuvant radiation for PASC [24, 31].

Several limitations should be acknowledged, particularly considering the retrospective nature of the analysis. Some limitations were inherent to the design of the NCDB, including lack of information on chemotherapy regimen, radiation treatment details, percentage squamous histology, genomic alterations and other squamous differential drivers, as well as anatomic resectability staging. The exact reasons patients were selected for preoperative therapy or SF are multifactorial, unmeasured, and could bias the results. Importantly, this retrospective study is not intention to treat. Only patients who underwent surgical resection could be included. Since not all patients who initiate NT will undergo surgical resection, these findings should be interpreted accordingly [25, 32]. Finally, the overall sample size was limited, particularly for NT subgroup analysis, increasing the likelihood of type II errors.

Notwithstanding these limitations, among patients with localized PASC, the receipt of NT prior to surgical resection was associated with improved OS outcomes. Future research is needed to clarify the optimal neoadjuvant treatment regimen, including the role of preoperative radiation, to enhance response to therapy and optimize short- and long-term outcomes for patients with this aggressive malignancy.

## Data Availability

The data that support the findings of this study are openly available in the National Cancer Database at https://www.facs.org/quality-programs/cancer/ncdb.
